# Progression of incomplete toward complete left bundle branch block: A clinical and electrocardiographic analysis

**DOI:** 10.1111/anec.12732

**Published:** 2019-12-11

**Authors:** Ellie Senesael, Simon Calle, Victor Kamoen, Roland Stroobandt, Marc De Buyzere, Frank Timmermans, Jan De Pooter

**Affiliations:** ^1^ Department of Cardiology University Hospital Gent Gent Belgium

**Keywords:** complete left bundle branch block, incomplete left bundle branch block, QRS notching

## Abstract

**Background:**

Complete left bundle branch block (cLBBB) is associated with increased cardiovascular mortality and heart failure. On the contrary, the clinical relevance of incomplete left bundle branch block (iLBBB) is less known. This study investigated the profile and outcome of iLBBB patients and assessed the risk of progression to cLBBB.

**Methods:**

Patients diagnosed with iLBBB between July 2013 and April 2018 were retrospectively included. Subsequently, echo‐ and electrocardiographic examinations at time of iLBBB diagnosis and during follow‐up, as well as progression to non‐strict cLBBB and strict cLBBB, were evaluated.

**Results:**

The study enrolled 321 patients (33% female, age 74 ± 11 years). During the follow‐up of 21 (8;34) months, 33% of iLBBB patients evolved to non‐strict cLBBB and 27% to strict cLBBB. iLBBB patients who evolved to non‐strict or strict cLBBB were older, had more frequently reduced left ventricular ejection fraction, and had more often QRS notching/slurring in the lateral leads and inferior leads, compared to patients without progression to cLBBB. In multivariate analysis, only QRS notching/slurring in the lateral leads was independently associated with progression to non‐strict cLBBB (odds ratio 4.64, *p* < .001) and strict cLBBB (odds ratio 9.6, *p* < .001). iLBBB patients with QRS notching/slurring had a progression rate to non‐strict cLBBB of 52% and 49% to strict cLBBB.

**Conclusion:**

Among patients with iLBBB, up to one third of the patients progress to cLBBB within a period of 2 years. The presence of QRS notching/slurring in the lateral leads during iLBBB was the strongest predictor for progression toward cLBBB.

## 1 INTRODUCTION

Complete left bundle branch block (cLBBB) is associated with increased cardiovascular mortality, sudden cardiac death, and heart failure (Surkova et al., 2017). Therefore, the presence of cLBBB on the electrocardiogram (ECG) raises clinical awareness and often warrants further cardiac investigations and clinical follow‐up. Incomplete LBBB (iLBBB) is most often defined by a QRS morphology reminiscent of cLBBB, but with a QRS duration (QRSD) <120 ms (Surawicz, Childers, Deal, & Gettes, 2009). The clinical profile and natural history of patients with iLBBB are poorly investigated and remain therefore largely unknown (Willems et al., 1985). This study aims to assess (a) the clinical profile of iLBBB patients, (b) the rate and risk factors of progression to cLBBB, and (c) the outcome of iLBBB patients.

## 2 METHODS

### 2.1 Patient selection and iLBBB definition

The study enrolled all adult in‐ and outpatients diagnosed with iLBBB on standard twelve‐lead ECG at the Gent University Hospital between July 2013 and April 2018. The study was approved by the Ethics Committee of the Gent University Hospital.

Patients with suspicion of iLBBB diagnosis were screened by scanning the hospital digital ECG database (Muse Cardiology Information System, GE Healthcare) using the following criteria: (a) QRSD ≥110 and <120 ms; (b) negative QRS complex in leads V1 and V2; (c) absence of q waves in leads I, V5, and V6 (any of two); and (d) R‐wave peak time >60 ms in leads I, aVL, V5, and V6 (any of two) (GE Healthcare, 2008).

Subsequently, all ECGs were visually analyzed. iLBBB diagnosis was manually confirmed by two independent cardiologists according to compliance to the American Heart Association (AHA) criteria (Table 1): (a) QRSD ≥110 and <120 ms; (b) R‐wave peak time >60 ms in leads V4, V5, and V6; and (c) absence of q waves in leads I, V5 and V6 (Surawicz et al., 2009). In case of borderline QRSD, measurements were manually confirmed using digital calipers. When multiple ECGs were available within a short follow‐up time, iLBBB diagnosis was withheld if confirmed on sequential ECGs.

**Table 1:** Electrocardiographic criteria to diagnose incomplete and complete LBBB^a^

Criteria	QRS duration	Additional features
iLBBB	110–119 ms	R‐wave peak time > 60 ms in leads V4, V5, and V6 Absence of q waves in leads I, V5, and V6
Non‐strict cLBBB	≥120 ms	QS or rS in lead V1 Monophasic R wave with the absence of q waves in leads V5 and V6
Strict cLBBB	≥120 ms	QS or rS in lead V1 Broad notched or slurred R wave in leads I, aVL, V5, or V6 Absence of q waves in leads V5 and V6

Note

Abbreviations: cLBBB: complete left bundle branch block; iLBBB, incomplete left bundle branch block.

a Surawicz et al., 2009; Surkova et al., 2017; Brignole et al., 2013.

### 2.2 Electrocardiographic analysis

ECGs were recorded at a paper speed of 25 mm/s and a calibration of 10 mm/mV with MAC 5,500 ECG recording devices (GE healthcare). ECG characteristics were digitally analyzed by the 12SL algorithm (GE Healthcare) including QRSD; maximum R‐wave amplitude and R‐wave peak time (lateral leads); QRS axis; and PR, QT, and QTc duration. Digital ECG measurements by this 12SL algorithm in patients with bundle branch block have been previously validated by our group (De Pooter, El Haddad, Stroobandt, De Buyzere, & Timmermans, 2017). The presence of QRS notching and slurring in the lateral and inferior leads was assessed by two independent investigators, experienced in ECG‐reading.

### 2.3 Progression toward cLBBB

Progression to cLBBB was assessed on follow‐up ECGs by two independent ECG readers. Assessment of progression toward cLBBB in patients with valvular disease was considered prior to valvular surgery to exclude iatrogenic cLBBB. To define cLBBB, a non‐strict cLBBB definition (QRSD ≥120 ms, QS or rS in lead V1 and monophasic R wave with the absence of q waves in leads V5 and V6) (Surkova et al., 2017) and a strict cLBBB definition (QRSD ≥120 ms; QS or rS in lead V1; broad notched or slurred R wave in leads I, aVL, V5, or V6; and absence of q waves in leads V5 en V6) (Brignole et al., 2013) were used (Table 1).

### 2.4 Echocardiographic and dyssynchrony assessments

Echocardiographic examinations within a 3‐month window of first iLBBB diagnosis were used for echocardiographic analysis. Left ventricular dimensions were measured in conventional parasternal views: left ventricular end‐diastolic diameter (LVEDD) and left ventricular end‐systolic diameter (LVESD). Left ventricular mass (LVM) was calculated using the Devereux formula (Lang et al., 2015). LVEDD, LVESD, and LVM were indexed for body surface area (BSA). The left ventricular ejection fraction (LVEF) was judged as normal (≥55%), mildly reduced (45%–54%), moderately reduced (35%–44%), and severely reduced (<35%).

Mechanical dyssynchrony was assessed by the presence of septal flash (SF). SF refers to a pre‐ejection leftward motion of the septum, followed by septal rebound stretch due to contraction of the lateral left ventricular wall and is considered a typical pattern of cLBBB‐induced mechanical dyssynchrony (Smiseth, Russell, & Skulstad, 2012). Two echocardiographic experts, blinded to the ECGs, reviewed all echocardiographic studies offline (EchoPAC version 7.1.13 and Xcelera viewer R3 version 3.3.1). The presence of SF was assessed visually and by M‐mode in apical window and parasternal long axis and short axis. This visual assessment of SF has previously been validated with low inter‐ and intra‐observer variability (Corteville et al., 2017).

### 2.5 Statistical analysis

Categorical variables are expressed as absolute number (percentage). Continuous variables are expressed as mean ± standard deviation in case of Gaussian distribution or median (1st and 3rd quartile) if data are non‐Gaussian distributed. Normality was tested using the Shapiro–Wilk test. To compare means of two variables, Student's *t* test and Mann–Whitney *U* test were used. Comparison of categorical variables among groups was performed by chi square test. Significant and near significant variables in univariate analysis were subsequently tested in a multivariate analysis using multiple logistic regression. Odds ratios (OR) are expressed with (95% confidence interval). Statistical significance was set at a 2‐tailed probability level of <.05. All statistical analyses were performed using SPSS software (version 25.0, IBM).

## 3 RESULTS

### 3.1 Clinical, electro‐, and echocardiographic characterization of iLBBB patients

The study enrolled 321 patients diagnosed with iLBBB on a standard twelve‐lead ECG. Mean age of the patients was 74 ± 11 years, and 33% of the patients were female. Coronary artery disease was present in 143 (45%) and valvular heart disease in 73 (23%) patients. Median QRSD at iLBBB diagnosis was 112 (110;116) ms. QRS notching and slurring in the lateral and inferior leads were observed in 123 (38%) and 82 (26%) iLBBB patients, respectively. Echocardiographic studies within a 3‐month window of iLBBB diagnosis were available in 243 patients. Mean LVEDD was 54 ± 9 mm, and 119 (51%) of the patients had a normal LVEF. Of interest, SF was detected in only 6 (2.5%) of iLBBB patients. Clinical, electro‐, and echocardiographic characteristics of all iLBBB patients are summarized in Table 2.

**Table 2:** Clinical, echo‐, and electrocardiographic characteristics of all iLBBB patients

	All patients (*n* = 321)
Clinical characteristics	
Age (years)	74 ± 11
Female sex *n* (%)	105 (33)
Length (cm)	170 ± 10
Weight (kg)	79 ± 16
BMI (kg/m^2^)	28 ± 5
BSA (m^2^)	1.90 ± 0.20
Systolic BP (mmHg)	124 ± 33
Diastolic BP (mmHg)	63 ± 17
Heart rate (beats/min)	73 ± 20
Underlying heart disease	
Ischemic heart disease *n *(%)	143 (45)
Congenital heart disease *n *(%)	9 (3)
Valvular heart disease *n *(%)	73 (23)
Heart failure *n *(%)	78 (24)
Hypertension *n *(%)	58 (18)
No overt heart disease *n* (%)	57 (18)
Echocardiographic measurements	
EDD (mm)	51 ± 9
EDD/BSA (mm/m^2^)	28 (25;33)
ESD (mm)	34 ± 11
ESD/BSA (mm/m^2^)	19 (15;24)
LVM (g)	213 ± 75
LVM/BSA (g/m^2^)	117 (91;156)
LVEF	
Normal (≥55%)	119 (51)
Mildly reduced (45%–54%)	56 (24)
Moderately reduced (35%–44%)	35 (15)
Severely reduced (<35%)	22 (10)
ECG measurements	
QRS duration (ms)	112 (110;116)
PR interval (ms)	180 (161;208)
Max R‐wave amplitude (µV)	712 (498;971)
QRS axis (°)	9 (−23;43)
QT interval (ms)	416 (386;448)
QTc interval (ms)	450 ± 39
Notching lateral leads *n*(%)	123 (38)
Notching inferior leads *n*(%)	82 (26)

Abbreviations: BMI, body mass index; BP, blood pressure; BSA, body surface area; EDD, end‐diastolic diameter; ESD, end‐systolic diameter; LVEF, left ventricular ejection fraction; LVM, left ventricular mass.

### 3.2 Progression rate to cLBBB

**Figure 1:**
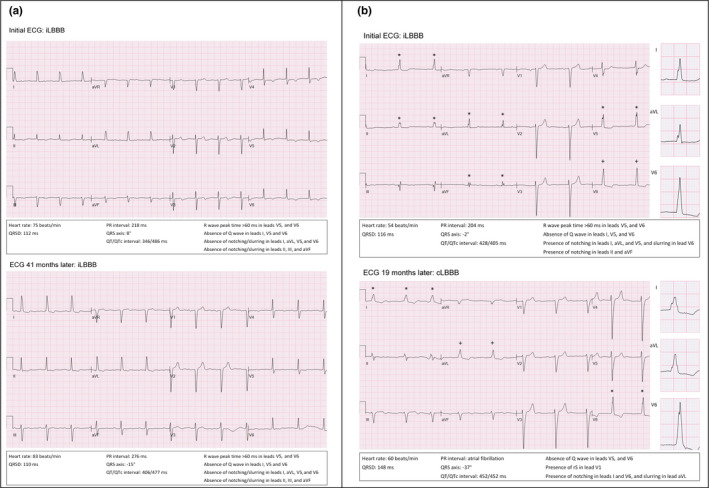
(a) Electrocardiograms from a 79‐year‐old female iLBBB patient (no QRS notching or slurring in the lateral and inferior leads) without progression to cLBBB during a follow‐up period of 41 months. (b) Electrocardiograms from a 74‐year‐old male iLBBB patient (QRS notching and slurring in the lateral and inferior leads) with progression to strict cLBBB during a follow‐up period of 19 months. QRS notching is marked by an asterisk (*) and QRS slurring by a plus sign (^+^)

Sequential ECG recordings were available in 215 patients with a median follow‐up period of 21 (8;34) months. Out of 215 patients, 72 (33%) patients showed progression from iLBBB to non‐strict cLBBB and 57 (27%) patients evolved to strict cLBBB. Representative ECG tracings are shown in Figure 1. Of interest, in only 6 (3%) patients, recovery to normal QRS was observed.

#### 3.2.1 Clinical predictors of progression toward cLBBB

iLBBB patients who evolved to non‐strict or strict cLBBB were older (75.8 ± 9.6 and 76.4 ± 9.6 years) compared to patients without progression to cLBBB (71.9 ± 11 years, *p* = .007 and *p* = .016, respectively). No differences in gender distribution, anthropometric characteristics, or underlying heart disease were detected between patients with and without progression toward cLBBB. Comparison of all clinical characteristics between iLBBB patients with and without progression to strict or non‐strict cLBBB is summarized in Table 3.

**Table 3:** Comparison of clinical, echo‐, and electrocardiographic characteristics between iLBBB patients with and without progression to strict or non‐strict cLBBB

	No progression to cLBBB	Progression to non‐strict cLBBB				Progression to strict cLBBB			
	*n* = 143	*n* = 72	Univar^+^ *p*‐value	Multivar^+^OR	Multivar^+^ *p*‐value	*n* = 57	Univar^#^ *p*‐value	Multivar^#^OR	Multivar^#^ *p*‐value
Clinical characteristics									
Age (years)	72 ± 11	76 ± 10	.016*	1.03 (1.00;1.07)	.700	76 ± 10	.007*	1.04 (1.00;1.08)	.056
Female sex *n *(%)	47 (33)	22 (31)	.732			17 (30)	.677		
Length (cm)	170 ± 10	169 ± 9	.203			169 ± 9	.367		
Weight (kg)	80 ± 17	80 ± 13	.143			79 ± 13	.283		
BMI (kg/m^2^)	28 ± 6	28 ± 5	.345			28 ± 5	.449		
BSA (m^2^)	1.91 ± 0.21	1.91 ± 0.16	.207			1.89 ± 0.17	.417		
Systolic BP (mmHg)	120 ± 38	129 ± 26	.191			131 ± 24	.122		
Diastolic BP (mmHg)	62 ± 20	65 ± 13	.681			66 ± 13	.814		
Heart rate (beats/min)	72 ± 19	71 ± 18	.521			71 ± 19	.647		
Underlying heart disease									
Ischemic heart disease *n *(%)	69 (63)	40 (53)	.155			33 (67)	.077		
Congenital heart disease *n *(%)	6 (5)	0 (0)	.084			0 (0)	.128		
Valvular heart disease *n*(%)	37 (28)	20 (32)	.616			16 (33)	.564		
Heart failure *n *(%)	37 (28)	22 (35)	.344			18 (37)	.271		
Hypertension *n *(%)	34 (25)	16 (25)	.934			15 (31)	.532		
Echocardiographic analysis									
EDD (mm)	50 ± 8	53 ± 9	.131			52 ± 9	.458		
EDD/BSA (mm/m^2^)	28 (24;38)	28 (25;32)	.982			28 (25;32)	.996		
ESD (mm)	31 ± 10	34 ± 11	.235			33 ± 11	.560		
ESD/BSA (mm/m^2^)	18 (15;22)	18 (13;24)	.866			18 (13;23)	.663		
LVM (g)	200 ± 66	228 ± 81	.066	1.00 (1.00;1.01)	.107	221 ± 79	.233	1.00 (1.00;1.01)	.194
LVM/BSA (g/m^2^)	116 (88;158)	121 (97;156)	.440			118 (96;159)	.513		
LVEF			.034*	0.47 (0.22;1.10)	.118		.154	0.67 (0.31;1.44)	.213
Normal (≥55%)	69 (59)	23 (41)				20 (45)			
Reduced (<55%)	47 (41)	32 (58)				24 (55)			
ECG measurements									
QRS duration (ms)	112 (110;114)	114 (110;118)	.021*	1.03 (0.94;1.13)	.510	114 (110;118)	.008*	1.04 (0.94;1.14)	.482
PR interval (ms)	180 (162;216)	183 (160;209)	.714			182 (158;208)	.697		
Max R‐wave amplitude (µV)	737 (498;976)	668 (518;960)	.457			668 (522;820)	.291		
QRS axis (°)	9 (−21;46)	6 (−29;38)	.257			2 (−28;35)	.189		
QT interval (ms)	420 (394;446)	410 (388;448)	0.504			410 (382;445)	.374		
QTc interval (ms)	450 ± 37	443 ± 39	.105			442 ± 40	.110		
Notching lateral leads *n *(%)	44 (31)	47 (65)	<.001*	4.6 (2.15;10.02)	<.001*	45 (79)	<.001*	9.60 (3.77;24.41)	<.001*
Notching inferior leads *n *(%)	29 (20)	24 (33)	.036*	0.13 (0.55;2.83)	.593	20 (35)	.028*	1.05 (0.42;2.60)	.915

Note

Statistically significant *p*‐values (*p* < .05) are marked with an asterisk (*) and bold font. *p*‐Values and ORs comparing patients with progression to non‐strict cLBBB vs. patients without progression to cLBBB are marked with a plus sign (^+^). *p*‐Values and ORs comparing patients with progression to strict cLBBB vs. patients without progression to cLBBB are marked with a pound sign (**^#^**).

Abbreviations: BMI, body mass index; BP, blood pressure; BSA, body surface area; EDD, end‐diastolic diameter; ESD, end‐systolic diameter; LVEF, left ventricular ejection fraction; LVM, left ventricular mass; multivar, multivariate analysis; OR, odds ratio; univar, univariate analysis.

#### 3.2.2 Echocardiographic predictors of progression toward cLBBB

iLBBB patients evolving toward non‐strict cLBBB and strict cLBBB had more frequently a reduced LVEF (58% vs. 41%, *p* < .036 and 55% vs. 41%, *p* < .068, respectively). No large differences in echocardiographic left ventricular dimensions were observed between patients with and without progression to non‐strict cLBBB and strict cLBBB. Comparison of all echocardiographic characteristics between iLBBB patients with and without progression to strict or non‐strict cLBBB is summarized in Table 3.

#### 3.2.3 Electrocardiographic predictors of progression toward cLBBB

iLBBB patients evolving to non‐strict cLBBB and strict cLBBB had more often QRS notching/slurring in the lateral leads (65% vs. 31%, *p* < .001 and 79% vs. 31%, *p* < .001, respectively). Likewise, QRS notching/slurring in the inferior leads was more frequently observed in patients evolving to non‐strict cLBBB and strict cLBBB (33% vs. 20%, *p* = .036 and 35% vs. 20%, *p* = .028, respectively) compared to iLBBB patients without progression to cLBBB. Differences in QRSD between patients with and without evolution toward cLBBB were small (114 vs. 112 ms, *p* < .05). No differences in R‐wave peak time, QRS axis, PR, QT, and QTc intervals were observed between groups. Comparison of all electrocardiographic characteristics between iLBBB patients with and without progression to strict or non‐strict cLBBB is summarized in Table 3. Of interest, among those who evolved from iLBBB to strict cLBBB, a significant difference in cLBBB QRS duration between females and males was observed (126 [123;129] ms vs. 130 [123;138] ms, respectively, *p* = .031).

#### 3.2.4 Multivariate analysis to predict progression toward cLBBB

In a multiple logistic regression model including QRS notching/slurring in the lateral and inferior leads, age, LVM, LVEF, and QRSD, only QRS notching/slurring in the lateral leads was independently associated with progression toward non‐strict cLBBB and strict cLBBB (OR 4.6 [2.15;10.02], *p* < .001 and OR 9.6 [3.77;24.41], *p* < .001, respectively) (Table 3). No collinearity was found among notching/slurring in the lateral and inferior leads (variance inflation factor <1.5).

### 3.3 Value of iLBBB QRS notching in the lateral leads

Out of 321 iLBBB patients, notching and/or slurring in the lateral leads (≥1 lead) was recorded in 123 (38%) patients. Patients with notching/slurring in the lateral leads were older (75 vs. 73 years, *p* = .029) and had more often concomitant notching/slurring in the inferior leads (37% vs. 18%, *p* < .001) and a small difference in QRS axis (3 vs. 16°, *p* = .029). Differences in clinical, echo‐, and electrocardiographic characteristics between iLBBB patients with and without QRS notching/slurring in the lateral leads are summarized in Table 4. Of interest, although the prevalence of SF among iLBBB patients was low, 5 out of 6 (83%) iLBBB patients with SF had QRS notching/slurring in the lateral leads. In female iLBBB patients with SF (3), lateral QRS notching/slurring was observed in all patients.

**Table 4:** Differences in clinical, echo‐, and electrocardiographic characteristics between iLBBB patients with and without QRS notching in the lateral leads

	Notching/slurring in lateral leads (*n* = 123)	No notching/slurring in lateral leads (*n* = 198)	*p*‐Value
Clinical characteristics			
Age (years)	75 ± 11	73 ± 11	.029*
Female sex *n *(%)	38 (31)	67 (34)	.585
BMI (kg/m^2^)	27 ± 4	28 ± 5	.360
BSA (m^2^)	1.88 ± 0.18	1.91 ± 0.22	.815
Echocardiographic measurements			
EDD/BSA (mm/m^2^)	28 (25;31)	28 (24;36)	.570
ESD/BSA (mm/m^2^)	19 (15;23)	19 (14;25)	.820
LVM (g)	219 ± 83	209 ± 70	.670
LVM/BSA (g/m^2^)	114 (96;159)	119 (89;151)	.634
LVEF			
Normal (≥55%)	46 (53)	73 (50)	.670
Mildly reduced (45%–54%)	20 (23)	36 (25)	
Moderately reduced (35%–44%)	15 (17)	20 (14)	
Severely reduced (<35%)	6 (7)	16 (11)	
ECG measurements			
QRS duration (ms)	112 (110;118)	112 (110;114)	.007*
Notching inferior leads *n *(%)	46 (37)	36 (18)	<.001*
PR interval (ms)	178 (160;204)	180 (162;209)	.468
Max R‐wave amplitude (µV)	615 (478;844)	805 (511;1008)	.002*
QRS axis (°)	3 (−29;38)	16 (−17;49)	.029*

Note

Statistically significant *p*‐values (*p* < .05) are marked with an asterisk (*) and bold font.

Abbreviations: BMI, body mass index; BSA, body surface area; EDD, end‐diastolic diameter; ESD, end‐systolic diameter; LVEF, left ventricular ejection fraction; LVM, left ventricular mass.

**Figure 2:**
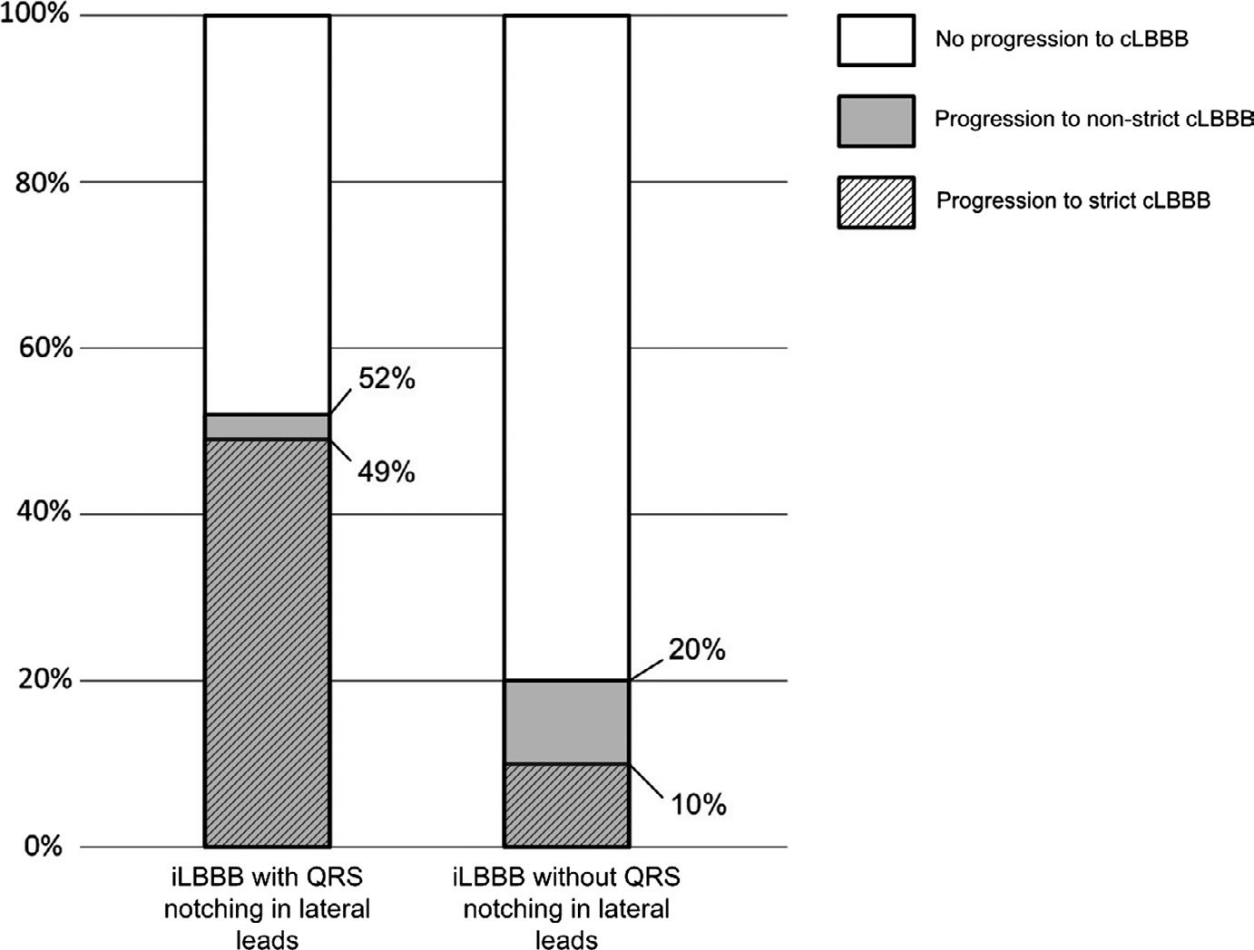
Evolution to cLBBB in iLBBB patients with and without QRS notching/slurring in the lateral leads

iLBBB patients with QRS notching/slurring had a progression rate toward non‐strict cLBBB of 52%, and 49% to strict cLBBB, indicating that most of the patients evolving to cLBBB fulfilled strict cLBBB criteria. However, in iLBBB patients without QRS notching/slurring, progression to non‐strict cLBBB was only 20% and 10% to strict LBBB, meaning that merely half of the patients evolving toward cLBBB fulfilled strict cLBBB criteria (Figure 2).

### 3.4 Outcome in iLBBB patients

Follow‐up data were available in 301 out of 321 patients. During a median follow‐up period of 31 (21;47) months, 101 (34%) patients died. None of the clinical, echo‐, or electrocardiographic parameters was independently of age associated with increased mortality.

### 3.5 Echocardiographic follow‐up in cLBBB patients

Follow‐up echocardiography was available in 44 out of 72 iLBBB patients evolving to non‐strict cLBBB. No significant differences were observed in LVEDD between iLBBB stadium and cLBBB stadium (53 ± 9.0 mm vs. 55 ± 9.2 mm, *p* = .437). LVEF decreased significantly after progression to cLBBB (percentage of patients with normal LVEF: 42% to 31%; patients with mildly reduced LVEF: 29% to 21%; patients with moderately reduced LVEF: 24% to 25%; patients with severely reduced LVEF: 6% to 23%; *p* = .008). When the analysis was restricted to patients evolving to strict cLBBB only, the same results were observed. SF was present in 67% of patients with strict cLBBB, whereas SF was not present in any of cLBBB patients not fulfilling the strict cLBBB definition (*p* = .009).

## 4 DISCUSSION

### 4.1 Main findings

To the best of our knowledge, this is the first study assessing progression from iLBBB to cLBBB. We show that among iLBBB patients, 26.5%–33.5% of the patients reveal evolution to cLBBB, depending on whether a strict or non‐strict cLBBB definition is used, respectively. The presence of QRS notching/slurring during iLBBB is the strongest predictor for progression toward cLBBB, independent of cLBBB definition. As such, patients with iLBBB and QRS notching/slurring in the lateral leads represent a population at high risk for the development of cLBBB.

### 4.2 History of defining iLBBB

Incomplete bundle branch block was first described in 1917 by Rothberger and Winterberg in canine studies (Rothberger & Winterberg, 1917). The concept of iLBBB was further elaborated by experimental animal and human studies of Sodi‐Pallares in the 1950s (Rodriguez & Sodi‐Pallares, 1952; Sodi‐Pallares, Estandia, Soberon, & Rodriguez, 1950)*,* defining iLBBB as “the presence of slurring in the beginning of the ascending limb of the R wave in those leads that reflect the potential of the left ventricle (I, aVL, V5, and V6), as well as the absence of a Q wave in the same leads.” Initially, QRSD was considered unimportantly in diagnosing iLBBB, but gradually a QRSD varying from 80 to 110 ms was accepted as criterion (Leighton, Ryan, Goodwin, Wooley, & Weissler, 1967; Sodi‐Pallares et al., 1950). iLBBB was redefined in 1985 by the WHO/ISFC Task Force criteria for conduction disturbances using following diagnostic criteria: (a) QRSD 100‐120 ms; (b) absence of Q waves in I, V5, and V6; and (c) R‐wave peak time >60 ms in V5 or V6 (Willems et al., 1985)*.* This latter definition, which does not require QRS notching or slurring, is still used today (Brignole et al., 2013; Surawicz et al., 2009).

### 4.3 The pathophysiology of QRS notching in the lateral leads

QRS notching/slurring has been proposed as diagnostic criterion to define “true cLBBB” and differentiate cLBBB from QRS prolongation with cLBBB‐like pattern caused by left ventricular hypertrophy (Strauss, Selvester, & Wagner, 2011). Several cLBBB definitions and guidelines on conduction disorders consider mid‐QRS notching/slurring as a key feature to diagnose “true cLBBB” (Brignole et al., 2013; Surawicz et al., 2009). According to the work of Strauss et al., QRS notching during cLBBB represents slowing of the right to left septal conduction, which occurs typically in cLBBB (Strauss et al., 2011). In electromechanical experiments in dogs with varying degrees of mechanically induced iLBBB, reversal of septal activation (right to left activation) could be documented if sufficient degree of iLBBB was accomplished (Rodriguez & Sodi‐Pallares, 1952). On the surface ECG, this reversal of septal activation was associated with widening of the QRS complex, disappearance of q waves in the lateral leads, and appearance of notching and/or slurring in the lateral leads. Minor degrees of iLBBB caused only a delay in left ventricular activation but did not change the left‐to‐right septal depolarization front, nor revealed the above mentioned ECG features. Identical electrocardiographic changes were observed during progressive impairment of left bundle branch conduction with increasing heart rates in patients with rate‐dependent iLBBB (Barold, Linhart, Hildner, Narula, & Samet, 1968; Schamroth & Bradlow, 1964).

### 4.4 Evolution of iLBBB to cLBBB

Data on progression from iLBBB to cLBBB are scarce, although one could assume that iLBBB might be a precursor of cLBBB. In this single‐center cohort study of iLBBB patients, we showed that up to one third of iLBBB patients evolved to cLBBB during a median follow‐up of 21 months. However, among iLBBB patients with QRS notching in the lateral leads, progression toward cLBBB occurred in half of the patients, whereas only 10%–20% of the patients evolved to cLBBB when QRS notching was absent. Therefore, from a clinical point of view, the presence of QRS notching identifies a population at high risk for evolution toward cLBBB. Whether this evolution to cLBBB translates into worse outcome needs to be further determined.

### 4.5 QRS notching/slurring as criterion to define “true” iLBBB

Our findings that QRS notching is associated with progression to cLBBB combined with the existing evidence that QRS notching in iLBBB is associated with reversed septal activation, raises the question whether QRS notching should be considered as a major diagnostic criterion to define “true” iLBBB. Indeed, the difficult electrocardiographic distinction between iLBBB and left ventricular hypertrophy has been a matter of debate since long (Willems et al., 1985). The presence of QRS notching/slurring might differentiate “true iLBBB” from QRS prolongation with iLBBB‐like pattern caused by left ventricular hypertrophy. Previous pathological work showed that 75% of iLBBB patients with presence of QRS notching in the lateral leads had truly injury to the proximal part of the left bundle branch, at its junction with the atrioventricular bundle (Unger, Greenblatt, & Lev, 1968). These pathological findings are in line with the growing evidence that most patients with “true” cLBBB, including the presence of QRS notching in the cLBBB definition, have a proximal lesion of the left bundle branch at the immediate exit of the bundle of His (Massoullie et al., 2016; Nguyen, Verzaal, Nieuwenhoven, Vernooy, & Prinzen, 2018; Sundh et al., 2015). In a recent study from Upadhyay et al., QRS notching was considered as the most distinctive ECG characteristic (highest sensitivity and best negative predictive value) to diagnose proximal cLBBB (Upadhyay et al., 2019). Most proximal cLBBB was correctable by His bundle pacing in this study, indicating that in those patients no distal conduction disease was present. As such, these findings confirm our results and might suggest that the evolution from iLBBB with notching to strict cLBBB reflects progressive impaired proximal conduction delay as explanation for the longer QRS duration.

Although SF was scarce in iLBBB patients, most of the patients with SF presented QRS notching during iLBBB. This is in line with previous work from our group showing that QRS notching during cLBBB is associated with SF among cLBBB patients (Corteville et al., 2017). The low prevalence of SF among iLBBB patients might indicate that these patients still have sufficient conduction in the left bundle branch (and therefore do not to exhibit SF).

Finally, all above‐mentioned findings suggest that iLBBB and cLBBB are entities within the same pathophysiologic spectrum of conduction delay in the left bundle branch and presumably only differ by the degree of impaired left ventricularconduction.

## 5 LIMITATIONS

Our population represents a hospital population and therefore both prevalence of iLBBB and progression to cLBBB in the general population might differ from our population. Given the retrospective study design, echocardiographic data were not retrieved for all patients and not all patients had paired data for clinical, echo‐, and electrocardiographic follow‐up variables, which may have limited our analyses. Especially, the assessment of SF was hampered by the limited availability of high‐quality echocardiographic data and the restricted echocardiographic follow‐up. Furthermore, we did not investigate the impact of clinical events during follow‐up on the natural history of progression from iLBBB to cLBBB. This could have given us a better understanding on when and why progression to cLBBB is to be expected. Follow‐up time was limited in our study. As one might assume that progression rates to cLBBB will be even higher during longer follow‐up, further investigation on outcome in iLBBB patients is needed.

## 6 CONCLUSION

In this single‐center registry of iLBBB patients, we showed that up to one third of patients reveal evolution to cLBBB during a median follow‐up of 21 months. The presence of QRS notching/slurring in the lateral leads during iLBBB was the strongest predictor for progression toward cLBBB, independent of the used cLBBB definition. As such, the presence of QRS notching/slurring during iLBBB on the twelve‐lead ECG identifies a population at high risk for the development of cLBBB.

## CONFLICT OF INTEREST

All authors declare that they have no conflict of interest.

## AUTHOR CONTRIBUTIONS

Authors contribution Senesael E: study design, data collection, data interpretation, manuscript drafting

Calle S: statistical analysis, data interpretation, manuscript drafting

Victor K: data collection, data interpretation

Stroobandt R: critical review

De Buysere M: Statistical analysis

Timmermans F: data interpretation, critical review

De Pooter J: study design, data interpretation, manuscript drafting

## ETHICS

The study was approved by the local Ethical committee of the University Hospital of Ghent.
